# Comparison between gas insufflation and gasless techniques for endoscopic transaxillary thyroidectomy

**DOI:** 10.3389/fendo.2024.1434419

**Published:** 2024-10-31

**Authors:** Li Lin, Shuxun Chen, Yizhuo Lu

**Affiliations:** ^1^ The School of Clinical Medicine, Fujian Medical University, Fuzhou, Fujian, China; ^2^ Zhongshan Hospital, Xiamen University, Xiamen, Fujian, China

**Keywords:** remote approach thyroid surgery, thyroidectomy, endoscopic, transaxillary, gas insufflation, gasless, thyroid tumor

## Abstract

**Objective:**

This study aimed to compare clinical outcomes and prognosis of endoscopic thyroidectomy via axillary approach using insufflation and gasless methods.

**Methods:**

Retrospective analysis included patients undergoing endoscopic thyroidectomy at our institution from June 2022 to October 2023. Patients were categorized into insufflation and gasless groups. Analysis compared surgical time, blood loss, drainage volume, tube removal time, hospital stay, complications, pain score, and incision satisfaction.

**Results:**

73 patients (48 insufflation, 25 gasless) were analyzed. Insufflation technique showed significantly superior outcomes: shorter surgery duration, reduced drainage volume, earlier tube removal, shorter hospital stay, and higher incision satisfaction (all P < 0.05). Postoperative pain (VAS) was lower in insufflation group on first day, but no significant difference on seventh day. No significant differences in blood loss or complications were observed.

**Conclusion:**

Insufflation technique offers advantages over gasless method including shorter operation time, reduced drainage, earlier tube removal, and shorter hospital stays, with comparable outcomes in pain and incision satisfaction.

## Introduction

Papillary thyroid carcinoma (PTC) is a prevalent endocrine malignancy worldwide. Recent studies have shown a rise in the global incidence of thyroid cancer over the past twenty years (1). The primary treatment for thyroid cancer is surgery, but conventional open thyroidectomy (COT) often results in visible scarring on the neck, making it less preferred. Particularly for young women and Asian patients with a predisposition to scarring, the presence of any neck scars is deemed unacceptable for cosmetic reasons (2).

Various minimally invasive and remote surgical techniques have been developed to address cosmetic concerns, such as laparoscopy and robotics, allowing for intricate procedures through smaller incisions (3, 4). Endoscopic technology has a certain priority in terms of aesthetic effect and postoperative quality of life, so it is generally considered as a cosmetic advantage of benign tumors, follicular adenomas and other surgical methods (5, 6). However, as a new treatment method for patients with malignant tumors, the safety and radical treatment of surgical methods should be confirmed. The safety and radical effectiveness of endoscopic or laparoscopic surgery in the treatment of malignancies in other organs has been demonstrated, with no difference in recurrence rates and survival between traditional open surgery and endoscopic or laparoscopic surgery (7). However, many researchers are skeptical about the feasibility of endoscopic thyroid surgery for radical treatment of malignant tumors (8). In a report on the effectiveness of minimally invasive video-assisted thyroidectomy (MIVAT) in PTC, the results are very similar to traditional surgery in terms of integrity, and its authors argue that endoscopic surgery is superior to open surgery when there is local aggression (9).

Transaxillary approach endoscopic thyroid surgery is one of the most widely used methods (10–13), it hides the incision in the natural fold of the axillary skin, has better cosmetic results than other methods, and is more feasible in the identification of the recurrent laryngeal nerve and the parathyroid gland, as well as the manipulation of the upper pole of the thyroid (14). In transaxillary endoscopic thyroidectomy, inflatable or airless techniques are used to maintain operating space. The aeration technique, which requires a carbon dioxide gas catheter to maintain a constant air pressure, was first invented by Ikeda in 2000. He used a continuous CO2 gas injection method to create a workspace, but at first the working space was relatively small, and the operating field of view was easily disturbed by smoke from the use of ultrasound knives. Saline irrigation and aspiration can also cause the collapse of surgical space (15). Therefore, to address this problem, a gasless endoscopic surgery method using an external retractor via a transaxillary approach has been developed (16). The gasless technique uses an external retractor to lift the chest flap and neck muscles without the problem of gas-related complications. With the introduction of robotic surgery, the airless technique has become more prevalent to assist in the operation of surgical robots (17, 18). The inflatable technique remains popular among laparoscopic surgeons. Each method has its own advantages and drawbacks, and the selection of technique should be based on individual patient needs and surgical goals.

## Patients and methods

In order to compare the two surgical methods, the main subjects included in this study were PTC patients. In 2006, Lombardi et al. reported the safety and efficacy of endoscopic thyroidectomy and selective neck dissection in the treatment of low-risk papillary thyroid microcarcinoma (19). For endoscopic thyroidectomy as a treatment for thyroid cancer, the selection criteria of tumor patients are the most important. As a result, we have some limitations in our criteria for inclusion of patients. This is because we are concerned about the limitations and safety of endoscopic thyroidectomy. “Low-risk” PTC, represented by small thyroid nodules with a good prognosis, is considered a suitable indication for endoscopic thyroidectomy. In addition, PTC is commonly seen in younger people, who are often concerned about the cosmetic effects of surgery.

### Patients

The clinical data of patients who underwent endoscopic transaxillary lobectomy for PTC at our hospital from June 2022 to October 2023 were retrospectively reviewed. The two surgical methods in our hospital have been carried out for many years, and the technology is relatively mature. Different surgical methods are selected according to the patients’ own wishes, and the patients are divided into inflatable group and non-inflatable group according to different surgical methods. Among them, the experimental group was inflatable group, a total of 48 cases, and the control group was non-inflatable group, a total of 23 cases. The age of the patients ranged from 18 to 60 years, with an average age of 40 years. All endoscopic procedures were successfully performed without the need for conversion to open surgery. Informed consent was obtained from each patient, who also signed a written consent form. All surgeries were conducted by a skilled surgeon.

Inclusion and exclusion criteria: Inclusion criteria included having a single tumor limited to one thyroid lobe, with a maximum tumor diameter of ≤2cm as measured by ultrasound. Preoperative fine needle aspiration biopsy (FNAB) confirmed the diagnosis of PTC. Patients had no evidence of lymph node metastasis, no bleeding disorders, and had high cosmetic expectations for neck appearance. Exclusion criteria comprised patients with a body mass index exceeding 35 kg/m2 or having overdeveloped neck muscles, FNAB results indicating poorly differentiated pathologic types with evidence of external thyroid invasion or distant metastasis, a history of prior neck or axillary surgery, or radiotherapy, and comorbidities such as significant heart, lung, brain, or other systemic diseases that precluded general anesthesia.

### Operative methods

Preoperative preparation included standard examinations such as thyroid function testing, neck X-ray, and laryngoscopy to assess tracheal compression, vocal cord function, and recurrent laryngeal nerve function. The skin was prepared and surgical markings were made prior to the operation. The extent of thyroidectomy was determined based on the guidelines of the American Thyroid Association (20), taking into consideration the specific patient population. In this study, unilateral lobectomy with isthmus and prophylactic unilateral central region dissection were the chosen surgical approaches.

#### Inflation group

After intubation, the patient is placed in the supine position. Shoulders are elevated, and the head is slightly turned to the healthy side to slightly extend the neck. The affected arm is extended 90°, fully exposing the axilla. The operative field is routinely sterilized and draped. A 1 cm incision is made along the midline of the axilla on the affected side, and a skin retractor is used to expand the subcutaneous tissue around the incision. A Trocar is inserted and a rigid endoscope is guided, with CO2 being injected and maintained at 6mmHg pressure. Two additional 5mm incisions are made in the same axilla and two more Trocars are inserted, guiding the left and right side operating instruments. The pectoralis major muscle is identified and dissection is performed along the pectoralis major muscle and the subcutaneous soft tissue between the muscle and the anterior chest wall. The SCM is identified and the space is further dissected along the SCM clavicle and coracoid bone notch, freeing the sternocleidomastoid muscle and the anterior neck muscle group. A laparoscopic thyroid hook is used to elevate the anterior neck muscles with an endoscopic thyroid retractor. The thyroid gland tissue is exposed. During the dissection, the side hole inserted into the endoscope Trocar is opened to discharge smoke and improve visibility, and the endoscope lens can be soaked in hot water if necessary, so that the endoscope lens is easier to keep clean and clear, and the time of repeated wiping of the lens during the operation is shortened.

Using fine dissection instruments and forceps, the gland is dissected between the true and false capsules in the endoscope. The gland superior pole is freed, and the thyroid superior artery is separated. The ultrasonic shears are held close to the gland and the superior pole is transected, lifting the superior pole towards the abdominal side. The recurrent laryngeal nerve is identified and confirmed, and its dorsal side is dissected with the ultrasonic shears, and the thyroid middle vein is identified and transected with the ultrasonic shears. The separation continued to the lower pole, and the pathological thyroid tissue, including tumors, was completely removed. The thyroid isthmus was separated in front of the trachea by separation forceps and cut off by ultrasonic knife. The intraoperative recurrent laryngeal nerve monitoring instrument detected the nerve signal throughout the operation. During the operation, the parathyroid gland was distinguished and protected, and its blood supply was retained. If it could not be retained *in situ*, it could be removed and cut up, dissolved in saline, and implanted between the sternocleidomastoid muscle or forearm muscle. The lymph nodes were separated in front of the trachea, the thymus was exposed and preserved, and the lateral lymph nodes were separated along the muscle and carotid sheath. The central lymph nodes were lifted, the recurrent laryngeal nerve was exposed and separated, and the esophagus and nerve were protected. The central lymph nodes were cleaned.

The removed thyroid and lymph nodes were put into a tissue specimen bag, and were taken out through the axillary incision for pathological examination. The wound was washed, completely stopped bleeding, and the parathyroid blood supply and the integrity of the recurrent laryngeal nerve were checked again. A negative pressure drainage tube was inserted into the wound and taken out through the axillary incision. Finally, all surgical incisions were sutured layer by layer.

#### Non-inflation group

The patient’s position was similar to that of the inflation operation. A 4-5cm long incision was made at the axillary fold, and the subcutaneous tissue was separated from the surface of the pectoralis major fascia with a long-handled electric knife. The flap was pulled up with a special retractor and inserted into the endoscopy and ultrasound knife. Then a 0.5 cm incision was made next to the incision, and Trocar was inserted into the endoscopic grasper or separation forceps.

First, we exposed the SCM anatomical tunnel to the anterior neck area. Secondly, the space between the sternal bone and the clavicle head of SCM was found under endoscopy, the space was separated from the cricoid cartilage to the clavicle, the position of the retractor was adjusted, and the space was separated from the sternohyoid muscle and sternothyroid muscle to the depth after the chest bone was lifted, and then the space of the retractor was fully dissociated between the sternothyroid muscle and the thyroid gland, and the position of the retractor was adjusted again. The free anterior cervical band muscle and the SCM chest bone were pulled upward and fixed on the traction scaffold to fully expose the thyroid gland and complete the establishment of surgical space.

The surgical procedures and precautions of thyroidectomy were basically the same as those of the inflatable method.

### Outcomes measured

Demographic data and thyroid nodule characteristics were recorded. The perioperative conditions of the two groups were analyzed. The intraoperative conditions included the total operation time (from skin incision to closure) and the estimated intraoperative blood loss. The postoperative data also analyzed the postoperative drainage flow, drainage tube removal time, hospital stay, postoperative complications, pain score and cosmetic effect. Among them, postoperative complications mainly included superior laryngeal nerve injury, recurrent laryngeal nerve injury, hypocalcemia, incision infection, and incision edema. For postoperative pain control, oral non-opioid analgesics are prescribed by the attending physician and administered immediately after surgery. The standard visual Analogue Scale (VAS) was used to assess pain on the first and seventh days after surgery on a scale of 0 to 10, with 0 indicating no pain, 10 indicating severe pain, and the middle part indicating varying degrees of pain. The beauty score was evaluated by Digital Evaluation Scale (NRS), and the satisfaction was evaluated by integers ranging from 0 to 10. The higher the score, the better the beauty effect. All clinical outcomes were compared between the two groups.

The incidence of postoperative complications was recorded. In all cases, laryngeal sensation and vocal cord movement were routinely observed by the surgeon within 1 week after surgery. The patient’s postoperative tone drop and drinking water choking were recorded as upper laryngeal nerve injury, and those who did not recover after symptomatic treatment for more than 6 months were considered to be permanent upper laryngeal nerve injury. Vocal cord paralysis and hoarseness caused by temporary recurrent nerve injury usually return to normal within 3 to 6 months after surgery, and vocal cord paralysis lasting 6 months is considered to be permanent recurrent nerve injury. Transient hypocalcemia usually appears within 72 hours after surgery. The clinical manifestations are numbness or hand, hand and mouth around the patient, which can be relieved within 4 to 5 days. Hypoparathyroidism more than 6 months is defined as permanent hypocalcemia.

### Statistical analysis

Data were imported into SPSS26.0 statistical software for statistical analysis. Descriptive statistics are used to summarize the characteristics of the research object. Measurement data is expressed as mean with standard deviation or median with interquartile spacing, and counting data is expressed as rate (%). The T-test is used to compare the mean of continuous data between two groups, using a chi-square test for correlations between categorical variables. P < 0.05 indicated that the difference was statistically significant.

## Results

### Patient characteristics

The patients included in the study were PTC patients who underwent transaxillary endoscopic lobectomy in our hospital from June 2022 to October 2023. There were 73 cases, 48 of which were aerated and the other 25 were non-aerated. The clinical characteristics of the patients are shown in **Table 1**. There were no significant differences in demographics, body mass index (BMI), and thyroid lesion characteristics between the two groups.

**Table 1:** Patient characteristics of gasless and gas insufflation transaxillary endoscopic thyroid lobectomy.

	Gas insufflation (n=48)	Gasless (n=25)	P value
Age (mean±SD)	39.9±10.5	38.9±6.8	0.605
Gender			0.933
Male(%)	20.8	20	
Female(%)	79.2	80	
Body mass index (mean±SD)	21.1±1.4	20.7±1.3	0.214
Size of nodule (cm) (mean±SD)	1.1±0.4	1.2±0.4	0.543

### Clinical results

The operative time of aerated group was significantly shorter than that of non-aerated group (59.2 ± 3.8 min Vs. 72.3 ± 3.2 min; P <0.001), and there was no significant difference in intraoperative blood loss between the two groups (**Table 2**). In addition, the postoperative drainage volume in aerated group was significantly lower than that in non-aerated group [95.0 (90.0,100.0) mL vs. 130 (125,137.5) mL; P < 0.001], and the drainage tube removal time was lower than that of the control group [3.0 (3.0,3.0) days vs. 4.0 (4.0,4.0) days; P < 0.001]. Similarly, the total hospital stay in the aerated group was significantly less than that in the aerated group [3.0 (3.0,3.0) days vs. 5.0 (4.0,5.0) days; P < 0.001]. In terms of pain, there were significant differences in VAS scores between the two groups on day 1 after surgery [5.0 (5.0,5.0) vs. 6.0 (6.0,6.0); P < 0.001], but there was no significant difference in VAS scores on day 7 after surgery. In addition, complication rates were not comparable between the two groups. Finally, the NRS score of the aerated group was higher than that of the non-aerated group [8.0 (7.0,8.0) vs. 6.0 (6.0,7.0); P < 0.001], and the difference was statistically significant (**Table 3**).

**Table 2:** Operative period data of gasless and gas insufflation transaxillary endoscopic thyroid lobectomy.

	Gas insufflation (n=48)	Gasless (n=25)	P value
Operative time (min) (mean±SD)	59.2±3.8	72.3±3.2	<0.001
Estimated blood loss (mL) (mean±SD)	19.7±1.8	20.3±1.4	0.192

**Table 3:** Postoperative period data of gasless and gas insufflation transaxillary endoscopic thyroid lobectomy.

	Gas insufflation (n=48)	Gasless (n=25)	P value
Postoperative drainage volume [median (IQR)]	95.0(90.0,100.0)	130(125,137.5)	<0.001
Drainage tube removal time (days) [median (IQR)]	3.0(3.0,3.0)	4.0(4.0,4.0)	<0.001
Hospital stay (days) [median (IQR)]	3.0(3.0,3.0)	5.0(4.0,5.0)	<0.001
Postoperative day 1 VAS score[median (IQR)]	5.0(5.0,5.0)	6.0(6.0,6.0)	<0.001
Postoperative day 7 VAS score[median (IQR)]	2.0(2.0,3.0)	2.0(2.0,3.0)	0.596
Postoperative satisfaction with incision at 3 months[median (IQR)]	8.0(7.0,8.0)	6.0(6.0,7.0)	<0.001

No deaths were observed during the study period, and there were no serious complications, including postoperative heavy bleeding, permanent voice changes, or gas-related complications. Both groups of patients had a pathological diagnosis of well-differentiated papillary carcinoma, limited to the thyroid gland, and no lymph node metastasis was found in the central area lymph nodes after surgery, thus no completion thyroidectomies or secondary neck dissection were required.

## Discussion

In recent years, with the increase of life pressure and the change of dietary habits, the incidence of thyroid diseases, especially thyroid micro-papillary carcinoma (PTMC, meaning the papillary carcinoma with the largest diameter < 1cm) has been increasing year by year under the increasingly popular diagnostic technology (21). Traditional open thyroid surgery requires incision of the cervical white line during the operation, which will lead to adhesion of the neck skin. Some patients feel strange neck sensation and swallowing discomfort after the operation, and there are obvious disadvantages of neck scars, causing psychological trauma to the patients. With the development of minimally invasive and “remote” surgical methods, endoscopic thyroidectomy has gradually become accepted by most patients and surgeons. In recent years, many reports have reported the experience of endoscopic thyroid surgery for low-risk PTMC (19, 22). Endoscopic surgery has a smaller wound, and the location of the wound can be moved to the cosmetic area (23). Different incision sites were used, including the chest wall, armpit, breast, and submandibular area (14, 15, 24–26). For transaxillary endoscopic thyroidectomy, there are two ways to maintain the work space: carbon dioxide injection and mechanical retractor lifting procedures, both of which are commonly used by surgeons. We hypothesized that these different mechanisms for maintaining operational space during surgery would lead to different clinical outcomes.

In order to make the comparison between surgical techniques as accurate as possible, the PTC patients selected for endoscopic thyroidectomy through axillary approach were not only similar in basic clinical features, but also had no significant differences in thyroid nodule size. In addition, all surgeries are performed by an experienced surgeon.

In our study, the aeration group showed significant benefits of the aeration technique in terms of total time to surgery, postoperative pain, and aesthetic satisfaction 3 months after surgery. Analysis of the reasons showed that in the non-inflatable group, due to the larger flap area separated during the establishment of surgical space, and the need to timely adjust the position of the retractor, the time was longer. Indeed, non-aeration technology has the advantages of large surgical space, not affected by gas expansion, and can avoid the occurrence of complications such as gas embolism and hypercapnia (27). However, the aesthetic results of aerated surgery are better (28), compared to non-aerated techniques, the injected CO2 can create relatively less operating space for soft tissue stripping, the postoperative discomfort in the thoracic area is less (29), and it also prevents wound contraction. Therefore, aeration has better incision satisfaction and less postoperative pain than airless surgery (30). In addition, it is reported that the occurrence of high carbonization and its severity depend on the aeration pressure of CO2, which should not exceed 16 mmHg in order to avoid complications (31). According to the study of Ochiai et al. (32), the optimal CO2 pressure is 6mmHg, which will greatly reduce the occurrence of gas-related complications. Our approach to this problem was to use a separation rod to widen the surrounding subcutaneous space, and after blunt separation of subcutaneous tissue, the inflation pressure of 6mmHg was sufficient to maintain the surgical space, but also low enough to avoid absorbing large amounts of carbon dioxide through the subcutaneous tissue. It is worth mentioning that our study results showed that the VAS results of the two groups were comparable on the 1st day after surgery, but there was no statistical difference on the 7th day after surgery. This was due to the similar degree of anatomy and the same postoperative analgesia regimen in the two groups, and the good analgesia regimen resulted in the good control of postoperative pain in the two groups. In this study, there was no statistically significant difference in the estimated intraoperative blood loss (P > 0.05), which was due to the surgeons’ understanding of the anatomical level and surgical proficiency, regardless of the operation space maintenance technique.

In terms of postoperative related indexes, the postoperative drainage flow, drainage tube removal time and hospitalization days in the aeration group were significantly less than those in the non-aeration group. This may be due to the different mechanisms by which different workspace maintenance techniques exert pressure on soft tissue resistance during surgery (33). In the non-inflatable group, when external pulling equipment was used to maintain the surgical space, the pressure was directly acted on a specific soft tissue, and the stress on the soft tissue increased with the increase of the operative time. This not only produced a lot of drainage fluid, but also led to a longer hospital stay. However, in CO2 aeration technology, this pressure diffuses into the soft tissue surrounding the operating space, minimizing pressure on specific areas of the soft tissue. In addition, other factors such as the proficiency of surgical assistants and individual differences of patients may be the factors that determine the poor prognosis of the airless group.

There was no significant difference in the incidence of postoperative complications between the two groups (all P > 0.05). In the aeration group, 1 case (2.08%) had water choking, 2 cases (4.17%) had hoarseness and 1 case (2.08%) had hypocalcemia. In the airless group, 1 case (4.00%), 1 case (4.00%) of hoarseness and 1 case (4.00%) of hypocalcemia occurred after operation. No tone drop, incision infection or edema were observed in both groups. Postoperative injury of recurrent laryngeal nerve and internal branch of superior laryngeal nerve was found in both groups. Postoperative injury of recurrent laryngeal nerve and internal branch of superior laryngeal nerve occurred in both groups, which may be caused by thermal injury caused by ultrasound activated scalpel during gland resection or excessive nerve pulling during operation (34). In this study, parathyroid glands were preserved *in situ* in both groups during the operation, and there was 1 case of hypocalcemia in each group after the operation, which was related to hypoparathyroidism. Some authors suggest that a single functional parathyroid is sufficient to maintain normal glandular activity. On the other hand, some authors believe that at least three parathyroids are needed to restore normal function (35). In AlqahtaniSM’s study (36), the occurrence of hypocalcemia in patients after unilateral thyroidectomy may be related to low preoperative calcium levels, parathyroid tissue loss, and postoperative decreased parathyroid hormone levels. The function of the above complications can be recovered after symptomatic treatment.

In summary, aeration technology has better clinical results than airless technology, but there are still some limitations worth noting. First, the patients we studied were followed for a short period of time after surgery because PTC can recurs even 20-30 years after thyroidectomy. However, two-thirds of recurrences typically occur in the first decade after the initial surgery, and especially in the first five years, the period of highest risk (37). In addition, the quality of life of cancer patients may gradually improve after surgery (38). In addition, we did not conduct statistical analysis of preoperative quality of life in the two groups. Second, differences in the abilities of surgeons for each surgical technique are difficult to eliminate, which can affect differences in outcomes. Finally, this study is a single-center, small-sample case study, and there are some limitations in the number of patients included and the criteria, which limit the reliability of the research results.

## Conclusion

Compared with the non-inflatable group, the inflatable group had a shorter surgical time, less acute postoperative pain, and better incision satisfaction. And because of gas injection, the inflatable group provided significantly better clinical outcomes in terms of postoperative drainage flow, drainage removal time, and length of hospital stay. There was no significant difference in the estimated intraoperative blood loss and the incidence of postoperative complications. Therefore, aeration via axillary approach complete endoscopic thyroid surgery has more advantages than non-aeration method and is worth popularizing.

## Data Availability

The original contributions presented in the study are included in the article/supplementary material. Further inquiries can be directed to the corresponding author.
